# Minimally invasive surgical technique for unstable supracondylar humerus fractures in children (Gartland type III or IV)

**DOI:** 10.3389/fped.2024.1352887

**Published:** 2024-04-24

**Authors:** Chang-Hyun Lee, Sung-Taek Jung, Chun-Gon Park, Joonyeong Kim, Gyo Rim Kang, Sungmin Kim

**Affiliations:** ^1^ Department of Orthopedic Surgery, Chonnam National University Medical School and Hospital, Gwangju, Republic of Korea; ^2^ Department of Orthopedic Surgery, Chonnam National University Hospital, Gwangju, Republic of Korea

**Keywords:** supracondylar fracture, humerus, children, Gartland type III, Gartland type IV, minimally invasive

## Abstract

**Background:**

Achieving and maintaining anatomical reduction during the treatment of pediatric humerus fractures, classified as Gartland type III or IV, presents a clinical challenge. Herein, we present a minimally invasive surgical approach using a novel and simple K-wire push technique that aids in achieving and maintaining anatomical reduction.

**Methods:**

We reviewed data of children receiving treatment for supracondylar fractures of the humerus at our hospital between January 2016 and December 2020. Patients were divided into two groups based on the method of treatment: Group 1 was treated with the K-wire push technique, and Group 2 was treated with the standard technique as described by Rockwood and Wilkins. The medical records and radiographic images were reviewed. In total, 91 patients with Gartland types III and IV fractures were included, with 37 and 54 patients in Groups 1 and 2, respectively.

**Results:**

The postoperative reduction radiographic parameters and Flynn scores at final follow-up were not significantly different between the two groups.

**Conclusion:**

The minimally invasive K-wire push technique for unstable supracondylar fractures in children is a safe and effective alternative for improving reduction. Using this technique, complications can be minimized, and the requirement for open reduction can be reduced.

## 1 Introduction

Supracondylar fractures are common pediatric injuries accounting for up to 60% of all elbow fractures in children (1, 2). The Gartland classification is widely used to guide the management of these fractures, with Gartland type III and IV fractures considered the most severe (3). Achieving and maintaining anatomical reduction in treating Gartland type III or IV supracondylar fractures can be significantly challenging because of multidirectional instability (4, 5). Due to the anatomical structure in which the medial and lateral columns are connected by thin segments, cortical contact is limited, making it challenging to maintain a stable reduction state (4).

To date, minimally invasive techniques using K-wires and mosquito forceps have been used to help reduce and maintain reduction (6, 7). However, these methods have several disadvantages, including the requirement of a stab wound and manipulation of the fracture site. Therefore, in this study, we aimed to introduce a minimally invasive surgical approach using a novel and simple K-wire push technique that aids in achieving and maintaining anatomical reduction of severely displaced supracondylar fractures of the humerus in children and to evaluate its clinical outcomes.

## 2 Materials and methods

### 2.1 Patients

We reviewed the records of 120 children who underwent treatment for supracondylar humeral fractures at our hospital between January 2016 and December 2020. Supracondylar fractures were classified according to the Gartland classification, and Gartland type IV fractures were classified based on intraoperative instability (3). Among them, 23 were classified as Gartland type II, 76 as Gartland type III, and 23 as Gartland type IV. Among the 99 patients with Gartland type III or IV fractures, 6 type III and 2 type IV patients were excluded from the study due to loss to follow-up within 1 year. In addition, patients who underwent open reduction were excluded. Finally, 91 participants with Gartland types III and IV fractures were included in this study.

Patients were divided into two groups based on the treatment method. Group 1 consisted of 37 patients who were treated with the K-wire push technique, while Group 2 included 54 patients who underwent the standard technique, previously described by Rockwood and Wilkins (8). The fractures were fixed by inserting two or three lateral pins. The criterion for using the K-wire push technique was used in Group 1 when satisfactory reduction maintenance was not achieved after three attempts at closed reduction.

In Group 1, two patients developed median nerve injuries preoperatively, and all 37 fractures were closed. In Group 2, four patients developed median nerve injuries and one patient experienced radial nerve injury. 53 fractures were closed, and one was open.

We reviewed the medical records and radiographic images of both the anteroposterior and lateral views, and obtained the average surgical time and postoperative reduction parameters including Baumann's angle, perpendicular distance, hourglass angle, and humerocondylar angle (8–11). The Flynn score was measured during outpatient follow-up (12).

This study was conducted in accordance with the Declaration of Helsinki and approved by the Institutional Review Board of the Chonnam National University Medical School and Hospital. The need for obtaining informed consent was waived owing to the retrospective nature of the study.

### 2.2 Techniques

**Figure 1:**
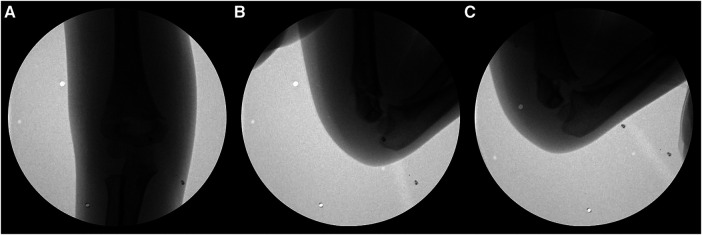
(**A**) Closed reduction was completed in the anteroposterior view. (**B,C**) When viewed laterally after closed reduction, the reduction could not be maintained due to instability, and there was a tendency of internal rotation of the distal fragment.

During surgery, the arm was prepared and draped, and closed reduction was attempted using an image intensifier under general anesthesia with the patient in the supine position. We subsequently attempted a closed reduction by traction of the arm, pronation or supination, and hyperflexion, and the lateral image was confirmed in a posture of external rotation of the shoulder and elbow flexion at 90°. When viewed laterally after closed reduction, the residual rotation tended to be an internal rotation of the distal fragment (Figure 1). Due to instability, reduction could not be maintained, and the distal fragment was internally rotated such that the medial side of the distal fragment was positioned posterior to the proximal shaft (Figures 1B,C). The K-wire push technique was used when maintaining reduction proved difficult after three attempts.

**Figure 2:**
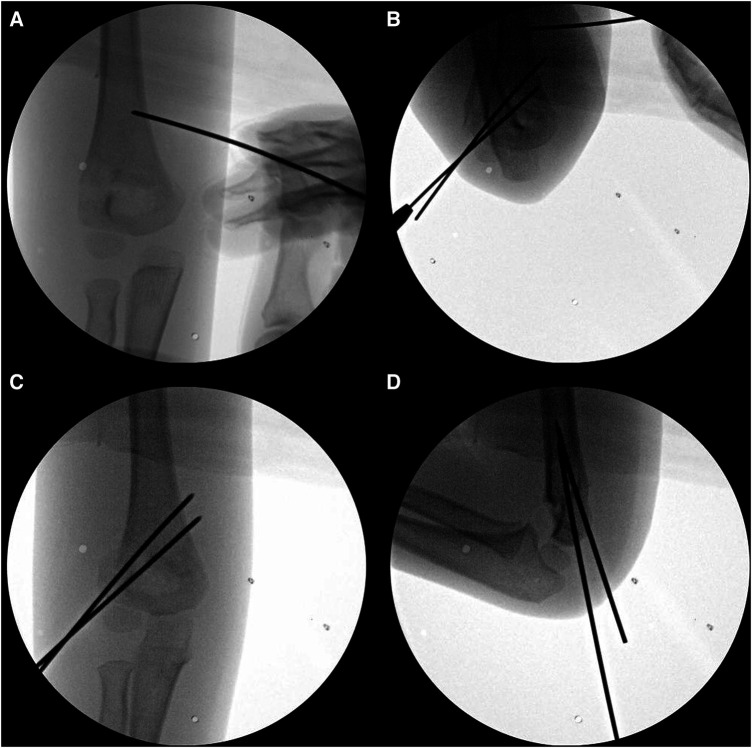
(**A**) In the K-wire push technique, the medial side of the proximal shaft fragment is percutaneously pushed from anterior to posterior with a 1.6 mm K-wire. (**B–D**) After confirming satisfactory reduction in the lateral view, repositioning of the shoulder to a neutral position was achieved and two 1.6 mm K-wires were inserted to secure stabilization.

**Figure 3:**
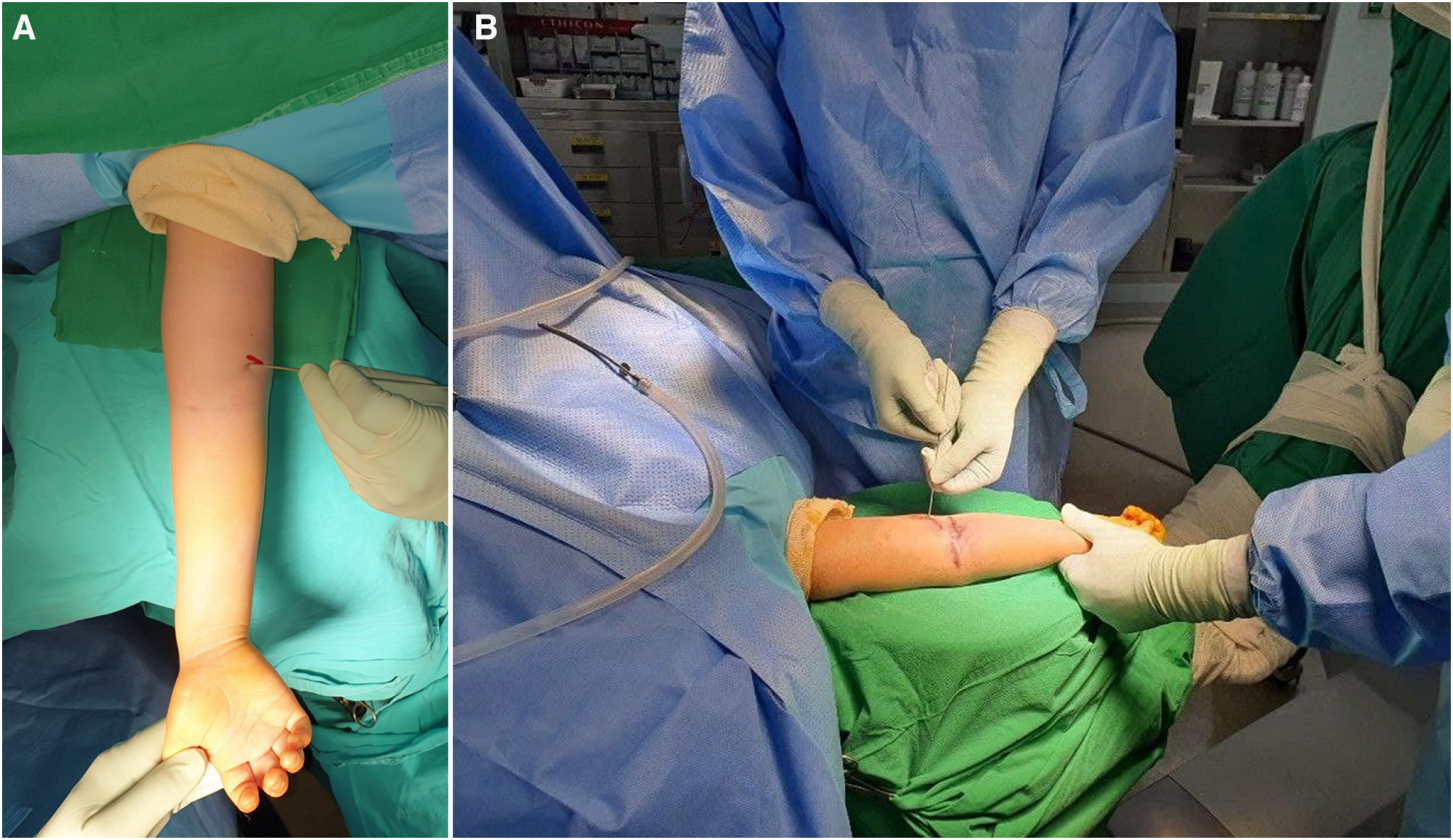
(**A,B**) Percutaneously, a 1.6 mm diameter K-wire was inserted obliquely at 45° from the axial plane to avoid nerve damage toward the target area identified by the image intensifier. Subsequently, the medial side of the proximal shaft fragment was pushed from the anterior to posterior direction, which helps maintain reduction for residual rotational malalignment and instability.

The K-wire push technique involves pushing the medial side of the proximal shaft fragment from the anterior to the posterior (Figure 2A). Percutaneously, a 1.6 mm diameter K-wire was inserted obliquely at 45° from the axial plane to avoid nerve damage toward the target area identified by the image intensifier. The initial step in skin pricking is not determined by palpating the landmark, but by placing a K-wire on the skin surface and checking it through the c-arm to estimate the depth. It is important to avoid causing damage to the brachial artery and median nerve when the insertion angle is too vertical. Similarly, if the insertion angle is too low, the bone cannot be pushed anatomically, and precautions should be taken to prevent damage to the ulnar nerve. The medial side of the proximal shaft fragment was then pushed in the anterior to posterior direction, which helps maintain a reduction in residual rotational malalignment and instability (Figure 3).

After confirming satisfactory reduction in the lateral view, the shoulder was turned neutral again and stabilized using two 1.6 mm K-wires (Figures 2B–D). If instability persisted, a lateral third pin or crossed medial pin was additionally inserted. Postoperatively, a cast was applied with the elbow flexed at 90° and the forearm in a neutral position. Outpatient follow-ups were performed at 2 weeks, 4 weeks, 2 months, 6 months, and 1 year after surgery, and every subsequent year thereafter. The K-wires were removed at the outpatient follow-up after 4 weeks. If incomplete bone union was observed, casting was continued for an additional week. Range of motion exercises were then initiated.

### 2.3 Postoperative evaluation and measurements

**Figure 4:**
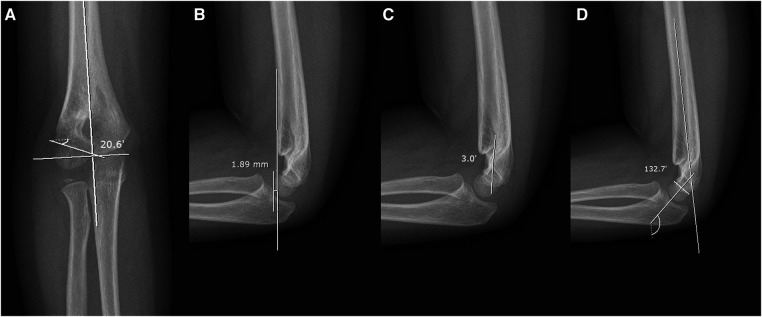
(**A**) Baumann angle. (**B**) Perpendicular distance from the anterior humeral line to the capitellum. (**C**) Hourglass angle. (**D**) Humerocondylar angle.

We reviewed the medical records and radiographic images of both the anteroposterior and lateral views. The Baumann angle, perpendicular distance from the anterior humeral line to the capitellum, hourglass angle, and humerocondylar angle were measured 4 weeks after pin removal (Figure 4) (8–11). The Bauman angle was defined as the angle between the line perpendicular to the longitudinal axis of the humerus and the line parallel to the growth plate of the capitellum (9). The perpendicular distance was determined by measuring the distance between the anterior humeral line and the furthest point on the anterior capitellum (9). In cases in which the capitellum was posteriorly displaced compared to the anterior humeral line, a negative value was documented. The hourglass angle was defined as the angle formed by the bisecting lines on the lateral radiographs between the olecranon and coronoid fossa, resembling the shape of an hourglass (8–10). The narrow neck of the hourglass served as the apex of this angle, which was determined from two lines positioned precisely in the middle of each side or the superior and inferior bulbs of the hourglass. The humerocondylar angle was defined as the angle between the axes of the distal humeral shaft and the distal humeral condyle, which bisects the capitellum into equal parts (11). All radiological reviews to confirm the measurements were performed by an author who was not involved in the clinical care of the patient.

### 2.4 Statistical analysis

The Statistical Package for Social Sciences (SPSS) (version 24.0) was used for statistical analysis. All continuous variables are expressed as means with standard deviations. All continuous variables were tested for normal distribution using the Shapiro–Wilk test. The Wilcoxon signed-rank test was used to compare the Baumann angle, perpendicular distance, hourglass angle, and humerocondylar angle between the two groups. Chi-square and Fisher's exact tests were used to analyze differences in Flynn scores.

## 4 Results

The mean age at operation was 5.5 (range, 3.0–12.3) years. The mean follow-up period was 33.9 months. The average surgical time was 47.97 (± 28.32) minutes in Group 1 and 44.91 (± 22.58) minutes in Group 2. In both groups, there were no cases of conversion to open reduction (Table 1). All fractures were of the extension type, and satisfactory reduction was obtained in all patients. Postoperative reduction parameters, including Baumann's angle difference, perpendicular distance, hourglass angle, and humerocondylar angle, were not significantly different between the two groups (Table 2). The percentage of good or excellent Flynn scores at the final follow-up also did not differ significantly. Complete bone union was observed in all patients. There were no cases of iatrogenic nerve injury. Postoperative complications occurred in two cases (2%). Groups 1 and 2 each had 1 case of superficial wound infection (2% and 1%, respectively). however, superficial wound infection resolved shortly after pin removal and administration of oral antibiotics.

**Table 1:** Patient demographics.

	Group 1 (K-wire push technique) *n* = 37	Group 2 (standard technique) *n* = 54	*P*
Male/female	22/15	32/22	
Age (years)	5.78 ± 2.88	5.59 ± 2.37	
Injured side (right/left)	16/21	28/26	
Gartland type (III/IV)	25/12	45/9	
Operation time (min)	47.97 ± 28.32	44.91 ± 22.58	0.401
Conversion to open reduction	0	0	
Follow up duration (months)	32.54 ± 15.43	34.72 ± 16.86	

**Table 2:** Comparison of radiographic parameters & clinical outcomes between groups 1 & 2.

	Group 1 (K-wire push technique) *n* = 37	Group 2 (standard technique) *n* = 54	*P*
Radiographic parameters			
Baumann angle (deg.)	21.5 ± 5.6	20.6 ± 4.8	0.340
Perpendicular distance (mm)	−0.5 ± 2.9	−0.7 ± 2.6	0.815
Hourglass angle (deg.)	169.5 ± 8.1	171.2 ± 8.5	0.148
Humerocondylar angle (deg.)	31.8 ± 10.5	32.5 ± 11.7	0.891
Clinical outcomes according to Flynn et al. (12)			
Cosmetic^a^ (%)	93.6%	95.7%	0.741
Functional^a^ (%)	91.0%	93.3%	0.713

^a^ Percentage of Flynn scores that were good or excellent.

## 5 Discussion

Achieving and maintaining anatomical reduction can be challenging in cases of severe and unstable supracondylar humeral fractures. Minimally invasive techniques have recently been developed to treat fractures that are difficult to reduce and maintain. In this study, we introduced a minimally invasive K-wire push technique that can maintain reduction. Our results indicated that there was no difference between Group 1, where maintaining reductions was challenging, and Group 2, where reductions were easily maintained within three attempts. The results demonstrate the effectiveness of the K-wire push technique in achieving and maintaining reduction in all cases, with no incidents of nerve injury or infection.

Lee et al. introduced a pin-leverage technique in which a pin was inserted into the fracture site via a posterior skin incision and used as a lever to attempt reduction of Gartland type III supracondylar fractures (6). When the clinical and radiographic results were compared among the three groups using open reduction, closed reduction with percutaneous pinning, and pin leverage techniques, the pin leverage technique was found to have good and comparable results when compared to other techniques. Li et al. previously reported that in extension type supracondylar fractures of Gartland type III, the insertion of mosquito forceps into the fracture site through an anterior skin incision to release interposed soft tissue or reduction through the lever technique was as effective and safe as the standard technique (7). The above two techniques can both be used to reduce irreducible supracondylar fractures; however, they have disadvantages such as complications in reaching the fracture site to manipulate the displaced distal fragment. The K-wire push technique introduced in the present study is a solution for reducible but rotationally unstable cases, and it allows convenient and easy manipulation of a proximal fragment that is relatively constant and easy to reach.

Novais et al. proposed the joystick technique to treat multidirectionally unstable supracondylar fractures and reported good results without complications (5). However, if the k-wire is inserted into the distal fragment as in the joystick technique, the process of inserting the k-wire for final fixation through the fracture will inevitably be hindered. Therefore, in irreducible cases, the joystick technique can be used to avoid open reduction. However, in reducible and unstable cases, the k-wire push technique is superior.

Leitch et al. proposed that after achieving reduction from the anterior to posterior view, the C-arm should be rotated during lateral imaging to prevent loss of reduction due to a high degree of instability (4). Rotational deformities were corrected by selectively pushing the lateral or medial condyles. However, it is not easy to achieve the lateral view by rotating the C-arm in a general operating room. A child's arm is smaller than that of an adult; therefore, it only protrudes slightly beyond the surgical bed, and there is a lack of sufficient space to rotate the C-arm by 90° for visualization. This can inevitably lead to contamination. In the present study, we introduced a minimally invasive technique for selectively pushing against residual rotational deformities in a more general C-arm setting.

In this study, we found no significant difference in the average operation time between Groups 1 and 2. On average, surgery in Group 1 took approximately 3 min longer. that the additional time was attributed to attempting the K-wire push technique when the reduction was not maintained well after three attempts at closed reduction, potentially prolonging the procedure. Therefore, the actual operation time did not differ between Groups 1 and 2. Lee et al. reported the average operation time, including anesthesia time, to be 68 and 57 min in the pin leverage and standard technique groups, respectively (6). Li et al. reported average operation times of 79 and 46 min in the minimally invasive and standard technique groups, respectively (7). This study did not include anesthesia time; therefore, the surgery time was slightly prolonged, likely influenced by the immediate application of the cast.

There were no cases of iatrogenic nerve injury in our study. The K-wire was inserted obliquely at 45° from the axial plane percutaneously into the anteromedial area of the antecubital fossa. At its location in the distal humerus, the median nerve is in front of the intermuscular septum, between the biceps brachii and brachialis muscles (13). Therefore, the 45° oblique approach can reduce the risk of median nerve injury, the bone can be palpated to safely and more accurately determine its location. The anterior approach was introduced by Carcassonne et al. in 1972, and according to Gennari et al., the fracture site is usually located just below the skin due to the ruptured brachial muscle (14, 15). Suh et al. previously reported that 78 surgeries using minimally invasive technique were successfully performed through an incision in the anteromedial part of the antecubital fossa, with no nerve injury reported (16). Suh et al. reported that nerve injury was avoided by performing blunt dissection. In most cases, blunt dissection was performed in minimally invasive techniques, and the incision size was 3 mm or more. The incision size was reported as 5 mm by Lee et al., 3–5 mm by Li et al., and 2 cm or 2–3 mm by Suh et al. (6, 7, 16). However, in the present study, as we used a 1.6 mm K-wire percutaneously, only a 1.6 mm pin-point wound was created.

The percentages of an excellent or good Flynn score were 93.6% and 95.7% for cosmetic factors and 91% and 93.3% for functional factors in Groups 1 and 2, respectively. In addition, clinical results were comparable to those of most previous studies (6, 7, 16). Lee et al. reported an excellent or good Flynn score in all patients, Suh et al. reported that in 97% patients, and Li et al. reported an excellent Mayo elbow index in all patients (6, 7, 16).

Superficial infection occurred in one patient each in Groups 1 and 2 (2% and 1%, respectively). However, the superficial wound infection resolved shortly after pin removal and administration of oral antibiotics. The infection rate in our study was comparable to the infection rate of 0%–4% reported in other studies on percutaneous pinning of supracondylar humerus fractures (17–20). Superficial wound infection rarely lead to serious septic arthritis or osteomyelitis, nor does it significantly impact functional outcomes (21). There were no cases of iatrogenic nerve injury and loss of reduction in our study. Conversely, Lee et al. reported superficial infection in four patients (4%) and iatrogenic nerve palsy in one patient (1%). In addition, Li et al. reported superficial infection in one patient (4%) and iatrogenic nerve injury in one patient (4%) (6, 7).

The primary limitation of this study was its retrospective nature with a relatively small sample size. Further, the cases treated with the K-wire push technique had more unstable fractures such as Gartland type IV fractures. This factor could have introduced selection bias, potentially influencing differences observed in the comparison between the two groups. Randomized, prospective studies may be needed in the future to confirm our findings. Nonetheless, this study is meaningful because the K-wire push technique group showed favorable outcomes comparable to those in the standard technique group, even with more complex fracture types.

## 6 Conclusions

In this study, the final clinical and radiological outcomes obtained with the K-wire push technique were similar to those obtained with standard closed reduction and percutaneous pin fixation. These results suggest that the minimally invasive K-wire push technique for treating unstable supracondylar fractures in children is an effective alternative to improve reduction with minimized complications.

## Data Availability

The raw data supporting the conclusions of this article will be made available by the authors, without undue reservation.
